# Sense of coherence, mental health, and hair cortisol concentrations among older people during the COVID -19 pandemic: a cross-sectional study

**DOI:** 10.1186/s12889-024-19034-3

**Published:** 2024-06-05

**Authors:** Jenny Koppner, Ann Lindelöf, Fredrik Iredahl, Staffan Nilsson, Annika Thorsell, Hanna Israelsson Larsen, Åshild Faresjö

**Affiliations:** 1 Department of Health, Medicine and Caring Sciences, Division of General practice, Linköping University, Linköping, Sweden; 2 Department of Health, Medicine and Caring Sciences, Division of Society and Health/Public Health, Linköping University, Linköping, Sweden; 3 Department of Biomedical and Clinical Sciences/Center for Social and Affective Neuroscience, Medicine, Linköping University, Linköping, Sweden

**Keywords:** Anxiety, Belief in the future, COVID -19, Depression, Economic status, Hair cortisol, Mental health, Older adults, Primary health care, Stress

## Abstract

**Background:**

A person’s sense of coherence (SoC) is likely to affect coping when exposed to a life changing event like the COVID -19 pandemic, which impacted the older population especially hard, an age group that already suffers from a lot of mental illness. Thus, the aim of this study was to investigate the associations between SoC and mental health in older adults using both screening scales and hair cortisol concentrations (HCC).

**Method:**

A cross-sectional design studying a cohort of 70–80 years old, N = 260, set in Swedish primary care during the pandemic years 2021–2022. Instruments used are sense of coherence 13 (SoC-13), EQ-5D-3L, Geriatric depression scale 20 (GDS-20), Hospital anxiety and depression scale (HADS), and Perceived stress scale 10 (PSS-10). Sociodemography and factors concerning SoC, and mental health are explored. HCC are measured using radioimmunoassay. Outcome measures are factors independently associated with SoC. Linear regression models were performed with SoC as dependent variable, and priory path analyses explored whether associations with SoC were direct, or indirect via anxiety.

**Results:**

SoC was significantly associated with anxiety (*p* < 0.001), perceived economic status (*p* = 0.003), belief in the future (*p* = 0.001), and perceived negative mental effect from the COVID -19 pandemic (*p* = 0.002). The latter was 96% indirectly associated with SoC (*p *< 0.001), whereas perceived economic status together with belief in the future was 82% directly associated with SoC (*p *= 0.17). HCC and sex were not significantly associated with SoC, but, noticeably, high HCC was equally distributed between women and men. Women reported significantly lower quality of life (*p* = 0.03), and more symptoms of anxiety (*p* = 0.001) and depression (*p* < 0.001).

**Conclusion:**

Anxiety, belief in the future, perceived negative effect on mental health due to the pandemic, and perceived economic status were significantly associated with SoC. Anxiety is suggested to be important in explaining the association between perceived negative mental effect from the COVID-19 pandemic and SoC. Women reported significantly poorer mental health and life quality than men.

**Supplementary Information:**

The online version contains supplementary material available at 10.1186/s12889-024-19034-3.

## Introduction

During the last decades there has been an increasing global trend of population aging, and by 2030 it is estimated that 1 in 6 people in the world will be aged 60 years or over, a number that will be doubled by 2050 [1]. With older age comes new challenges concerning social, physical, and mental health aspects, which will impact different aspects of society, e.g. healthcare, and community services. It is therefore of importance to face these challenges and facilitate healthy aging, not only for the individual, but also for society in general.

Sense of coherence (SoC) is a well-established concept sprung from the salutogenic theory introduced by Aaron Antonovsky in 1987. It focuses on what keeps a person healthy, opposed to why someone become ill, and comprises of the three key elements: 1) comprehensibility, 2) manageability, and 3) meaningfulness, which together have shown to possess health promoting effects regardless of age and sex [2, 3]. A Swedish study showed that SoC in the general population within a 10-year span (1994–2004) is relatively stable with minor changes that could be due to e.g., social changes [4]. Previous research from our group has compared the applicability of the SoC measurement between adults living in Sweden and Crete, in which Cretan women seem to score significantly lower than their Swedish counterparts, while Cretan men tend to report a higher SoC compared to their matched Swedish reference population [5]. Previous research on the older population has shown that SoC pools resistance resources and, thus, influences healthy aging positively [6]. It has also found that indicators of healthy aging have been positively associated with SoC, e.g. subjective wellbeing [7], good quality of life [8–10], and good mental and physical health [10, 11].

Challenging healthy aging could be extraordinary social circumstances like the recent COVID -19 pandemic, a major stressor [12] that interfered with most aspects of life and society. A high and stable SoC is likely to predict better coping in a stressful situation, nevertheless, it might not be enough when faced with a difficult life situation, depending on its severity. A Japanese study investigated the protective nature of SoC among older adults exposed to housing damage due to earthquake and following tsunami in 2011 [13]. They found that high SoC was protective against high mental distress when exposed to a smaller damage, but not when the exposure was more severe. Among the most vulnerable to the COVID -19 virus were the older adults, who, more than most others, needed to isolate and physically distance themselves from society [14, 15]. It is well known that the older population already is susceptible to poor mental health [16], women more so than men [17, 18], and the strains of the pandemic on this age group might have increased that burden even more. Present research on mental health among older adults during the pandemic is mainly from its first half and bring forward inconclusive data [19–23]. To this point, few published studies have investigated SoC and mental health during the pandemic. One such study on adults aged 20-95 years has been found showing SoC to be predictive of changes in psychopathological symptoms [24].

A pandemic can be considered a long-lasting stressor and as such possibly cause dysregulation of the hypothalamic–pituitary–adrenal (HPA) axis. This is a central biological mechanism in the interplay between chronic stress and mental distress for which hair cortisol concentrations (HCC) is a possible biomarker [10, 25]. Several studies have found that HCC increases with age and male sex [26, 27]. However, research exploring HCC and mental health in the older population from the pandemic years is scarce, but there is an Irish longitudinal study, which found higher HCC to be predictive of developing clinically significant depressive symptoms during the pandemic in older adults [28]. To our knowledge, there is also a general lack of research investigating SoC, HCC, and mental distress both in general and among older people. Nonetheless, at least one study was found investigating SOC and HCC among Palestinian adolescent victims of war. The result showed that HCC was significantly elevated in the Post traumatic stress disorder (PTSD) subgroup compared to the subgroup not exposed to trauma. SoC was inversely related to self-reported psychopathology, as well as to HCC in the trauma group [29]. A recent study proposes that the COVID-19 pandemic is a new type of traumatic stressor [30]. Considering the results in the previously mentioned study of the young [29] this emphasizes the importance to study the associations between the HPA-axis activity and SoC in the older population exposed to the COVID-19 pandemic.

As far as we know, there is a lack of research investigating SoC and mental distress from a primary care perspective among older people, both in general and during the COVID-19 pandemic. In Sweden almost all people are registered at a primary health care center (PHCC), regardless of needing medical attention or not. Thus, the Swedish primary care setting covers different socioeconomic groups, and incorporates both healthy and diseased during all later years in life, reflecting the general older population well.

Against this background, the aim of this study was to investigate the associations between sense of coherence and mental distress, analyzing both self-reflecting screening scales and biological stress, among older adults in a primary care setting during the extraordinary social circumstances of the COVID -19 pandemic.

## Materials and methods

### Participant recruitment

**Fig. 1:**
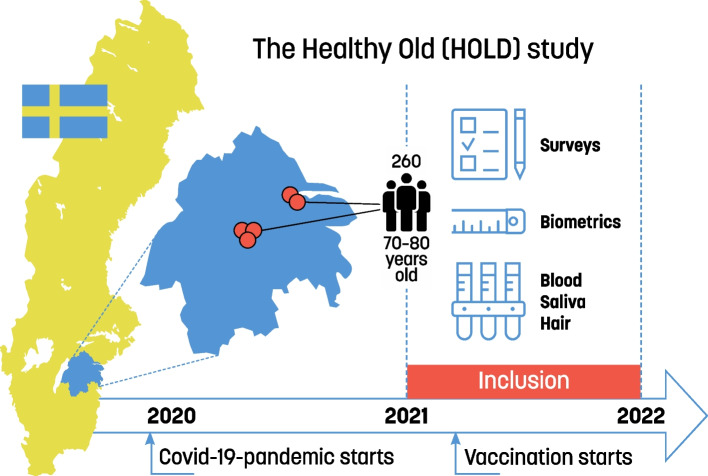
Overview of the HOLD-study protocol. In total 260 inhabitants in the Southeast of Sweden, between the age of 70–80 years, were recruited from five Primary Care Centers. At the inclusion subjects were studied in regards of biometric data, surveys, blood samples, hair sample, and saliva

This cross-sectional study is part of the Healthy OLD-study (HOLD), which focuses on the increasing older population in Sweden, investigating long-term stress, psychosocial and health factors, and Sense of coherence among 70–80-year-olds. In total, 260 participants were recruited from five PHCC in both rural and urban settings in the Southeast region of Sweden during the latter half of the COVID -19 pandemic, October 2021 – December 2022, as shown in Fig. 1. Each PHCC recruited approximately 60 participants (52-64) apart from one PHCC included later in the process and due its shorter recruitment period only recruited 29 participants. The population base constituted of all registered people in the age group at the five PHCC (*N* = 7796). Exclusion criteria were not finding the eligible participant’s phone number on public digital services, no answer during three call attempts, living in a nursing home, not being able to visit the PHCC, not having enough hair for the hair sample, inadequate Swedish language skills, and inadequate cognitive function. At each PHCC a list of all individuals in the age group was randomized. Thereafter, starting at the top of the randomized list, letters were sent out with written information, followed by a phone call from a research nurse who answered questions, investigated interest to participate, and assessed exclusion criteria. If all requirements were met and the person was interested to participate an appointment at the person’s PHCC was booked for signing of the written consent form, and data collection. If the person decided not to participate, he/she was excluded without having to give an explanation. The participant could at any point of time withdraw from the study. There were no dropouts once the written consent form was signed.

### Procedures and measures

The questionnaire was filled out during the PHCC visit where the research nurse was available to aid if needed. Included were commonly used and well validated instruments, as well as questions concerning the COVID -19 pandemic, and questions derived from The Swedish Living Conditions Survey of health and welfare, concerning sociodemographic factors, economic status, belief in the future, and perceived insomnia and self-perceived health [31]. To form the question regarding self-perceived health, the questionnaire also included a five-point-scale question about general health with ratings: 1 = excellent, 2 = very good, 3 = good, 4 = not so good, 5 = bad. For the analyses this was dichotomized into: 1–3 = good health, and 4–5 = poor health. To measure the participants sense of coherence and quality of life, the screening scales Sense of coherence 13-item (SoC 13), and EQ-5D-3L were used. SoC 13 measures to what extent a person feels confidence in their own ability to face adversity, which can be associated to wellbeing and health. The scale is based on three factors believed to be of importance for coping: the perception that existence is comprehensible, manageable, and meaningful. SoC-13 contains thirteen questions where every item is scored on a Likert scale ranging from 1 to 7 points. Thus, the total score is 13–91 points, higher scores indicate a more effective coping strategy, and better health outcome [2, 32]. This 13-item version of SOC has been shown to be reliable, valid, and cross-culturally applicable when evaluating how people can manage stress and still be healthy [11, 33]. A Swedish version of SOC-13 is used in this study [34]. To further study quality of life, the EQ-5D-3L was used. This assesses five dimensions of health: mobility, self-care, usual activities, pain and discomfort, and anxiety and depression, where higher scores reflect better quality of life [35]. For investigating mental health, the Geriatric Depression Scale 20-item (GDS-20), Hospital Anxiety and Depression Scale (HADS), and Perceived Stress Scale 10-item (PSS-10), all validated in Swedish, were used. The GDS-20 is a screening tool intended to identify depression in older people. It consists of twenty yes-or-no questions, 1 point given for each yes in the box with a total score of 0–20 points. Higher scores are associated with more likelihood of depression; 0–5 points depression is unlikely; 6–20 points depression is suspected [36]. Included are questions about high and low mood, lack of energy, anxiety, social withdrawal, and physical health [37], reflecting how the participant feels now and has felt during the last month. The Swedish version has been tested, and 70% with suspected depression could be confirmed using the diagnostic criteria "Diagnostic and statistical manual of mental health [36].

The HADS is a diagnostic tool consisting of two parts, one measuring depression (HADS-D) and the other measuring anxiety (HADS-A). Each part is made up of seven questions with a total score of 0–21 points, respectively, where the possibility of depression and/or anxiety disorder increases with higher scores [38]. The HADS is well validated and often used in studies, e.g. Bodlund et.al has used it in several studies, among others on 374 Swedish unselected primary care patients [39]. The HADS has a good reputation among general practitioners, psychiatrists, psychologists, and researchers [39]. The PSS-10 consists of ten questions on a Likert scale scoring 0–4 points, measuring how the participant have experienced stress during the last month. Total score is 0–40 points where higher scores indicate higher stress [40]. The Swedish version, as well as the original English version, have been demonstrated to be reliable and valid [41].

To add another dimension of stress, cortisol concentrations in hair was analyzed as an indicator of physiological stress. An approximately 3 mm thick hair sample from the vertex area of the head was cut as close to the skin as possible by trained staff, preferably at least three cm in length, but 1 cm was accepted if the hair volume was sufficient, in accordance with guidelines published the Society of hair Testing [42]. Hair in the vertex area of the head has a regular rate of growth [43]. A competitive radioimmunoassay (RIA) was used to extract and analyze cortisol concentrations, as described by Yang et al. [44, 45]. Briefly, the hair sample was cut into 3 cm pieces and measured with a ruler starting from the root. Samples weighing 5–7 mg were frozen for 2 min in liquid nitrogen and minced together with a steel ball in a Retch Cryo Mill (20 HZ) for 2 min. Methanol (1 ml) was added to each tube, and the samples were extracted overnight on a moving board. Then 0.8 ml of the methanol supernatant was pipetted off and lyophilized using a Savant Speed Vac Plus SC210A. The samples were dissolved in radioimmunoassay buffer and analyzed as described by Mörelius et al. [45]. The primary antibody used was a rabbit polyclonal antibody “Cortisol 3”, catalog number MBS535414 (MyBioSource, San Diego, USA). The secondary solid-phase antibody was anti-rabbit Sac-Cel (AA-Sac1, ImmunoDiagnostic Systems Ltd, Boldon, England). Previous work has shown that hair samples of 5 mg or more are needed to achieve an inter-assay coefficient of variation below 8% for hair extraction and cortisol measurement [46–48]. The intra-assay coefficient of variation for the radioimmunoassay was 7% at 10 nmol/L. HCC are expressed as pg cortisol/mg hair [46].

### Statistical analysis

**Fig. 2:**
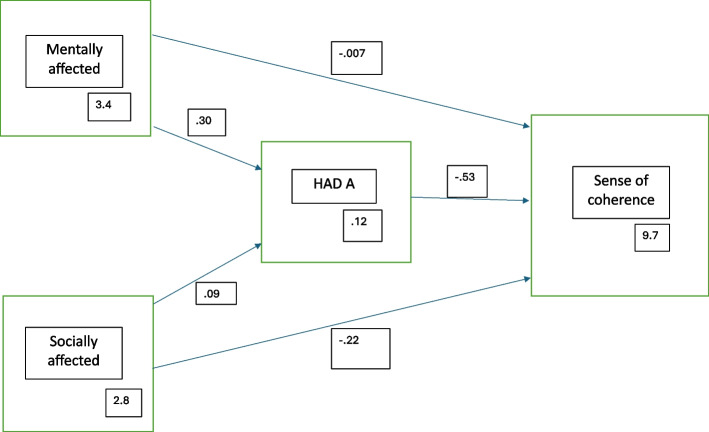
Structural equation model showing indirect or direct association with sense of coherence. Two variables are explored: *Mentally affected* = the variable perceived negative effect on mental health due to the COVID -19 pandemic: *Socially affected* = the variables perceived financial status together with belief in the future. HADS-A (anxiety) is used as a mediator (association expressed as *r*-value)

The SPSS version 28.0 software (SPSS Inc., Chicago, IL, USA) was used for statistical analysis, and a *p*-value of 0.05 was considered statistically significant. For descriptive statistics, including sex distribution in the different variables, bivariate tests were performed: in Table 1 Chi^2^ test comparing grouped variables was applied, whereas t-test was used in Table 2 as that displays variables treated as interval scales. Several of the screening scales do not have cut-offs why they in this study was treated as interval scales, using means and lowest/highest value to describe their distribution in the population. Cortisol concentrations in hair were divided into tertials for illustrative purposes in Table 1, but in all other analyses logarithmized values were used, reflecting more reliable data considering skewness in the data. Spearman’s correlation was used with SoC-13 as dependent variable compared to all other variables included in this study. A three-step regression analysis with SoC-13 as dependent variable was performed where model 1 consisted of sociodemographic factors, in model 2 psychosocial factors were added apart from mental health factors, which instead were included in the third model. Finally, a fourth regression model was analyzed including all statistically significant factors from model 3. To further explore the results in the final regression model a priori path model was fitted, as described in Fig. 2. Two variables were analyzed to investigate if these operate directly or indirectly, via anxiety as a mediator, with SoC. If a variable’s indirect path in the model account for a substantial fraction, there is a possibility that anxiety is important in explaining its association to SoC. In the model of this study the variable perceived negative effect on mental health due to the COVID -19 pandemic was labeled *mentally affected,* and the variables perceived financial status together with belief in the future built a new variable labeled *socially affected.*

**Table 1:** Characteristics of the total study population in relation to socioeconomic and psychosocial factors

**Variables**	**Male (*n* = 114)**	**Female (*n* = 146)**	***p*-value**
	**% (n)**	**% (n)**	
**Civil status**			0.017
Married/cohabited	79.6 (90)	66.2 (96)	
Single	20.4 (23)	33.8 (49)	
**Education**			0.95
Primary school	30.1 (34)	29.8 (42)	
Secondary school	33.6 (38)	35.5 (50)	
University/college	36.3 (41)	34.8 (49)	
**Perceived financial status**			0.16
Good	96.5 (110)	92.3 (132)	
Bad	3.5 (4)	51.4 (11)	
**Perceived social life**			0.54
Good	83.3 (95)	87.6 (127)	
Neither, nor	14.9 (17)	10.3 (15)	
Bad	1.8 (2)	2.1 (3)	
**Perceived health**			0.14
Good	90.4 (103)	84.1 (122)	
Bad	9.6 (11)	15.9 (23)	
**Perceived insomnia**			0.056
No	84.8 (95)	75.0 (105)	
Yes	15.2 (17)	25.0 (35)	
**Belief in the future**			0.17
Optimistic	67.6 (75)	57.2 (83)	
Neither or	28.8 (32)	35.2 (51)	
Pessimistic	3.6 (4)	7.6 (11)	
**Cortisol concentrations**			0.34
Low^a^	16.1 (18)	23.1 (33)	
Medium^b^	64.3 (72)	56.6 (81)	
High^c^	19.6 (22)	20.3 (29)	

^a^Tertial 1: 8,29–16,53 pg/mg

^b^Tertial 2: 16,84–61,25 pg/mg

^c^Tertial 3: 61.75–3445,64 pg/mg

**Table 2:** Characteristics of the total study population in relation to psychosocial factors and screening scales

**Variables**	**Male**			**Female**			***p*-value**
	**Mean (SD)**	**Lowest score**	**Highest score**	**Mean (SD)**	**Lowest score**	**Highest score**	
**SoC-13**	70.85 (8.59)	42	85	69.86 (8.32)	41.0	84.5	0.35
**EQ-5D**	0.83 (0.15)	0.193	1.000	0.77 (0.19)	0.088	1.000	0.030*
**PSS-10**	18.42 (2.88)	10	25	19.27 (3.10)	11	31	0.025*
**HADS-A**	3.68 (2.96)	0	13	5.00 (3.10)	0	14	0.001*
**HADS-D**	7.46 (1.31)	5	12	7.23 (1.04)	5	11	0.13
**GDS-20**	5.99 (1.67)	1	12	6.68 (1.87)	3	13	< 0.001*

T-test analysis was used for comparing means. Possible scores: *SoC 13* Sense of Coherence 13-item 13-91, EQ-5D 0-1.000, *PSS-10* Perceived Stress Scale 10-item, 0-40, *HADS-A* Hospital Anxiety and Depression Scale – Anxiety, 0-21, *HADS-D* Hospital Anxiety and Depression Scale – Depression, 0-21, *GDS-20* Geriatric Depression Scale 20-item, 0-20

^*^significant difference

## Results

The study involved 260 participants aged 70–80 years (mean age 75,12 ± 2.94; 56,2% females). As reported in Table 1, significantly more women than men lived as singles, more women than men, but not significantly, suffered from insomnia, and perceived their financial status as bad. The majority of the responders perceived good health, a good social life, and were optimistic for the future. HCC was equally distributed in women and men when viewing the tertials, see Table 1, but the median was slightly higher in men than women (24,56 and 23,41 respectively), however, neither difference was significant.

The principal psychological characteristics of the population as well as sense of coherence, and quality of life are summarized in Table 2; women scoring significantly lower quality of life, and higher anxiety and depression than men. Intercorrelations of the screening scales used in this study were significant with *p* < 0.001 or *p* = 0.004 for all but PSS-10 and EQ-5D (*p* = 0.24), and PSS-10 and HADS-D (*p* = 0.25), see Table S1.

Correlations were analyzed between sense of coherence and various factors, see Table 3. Inverse correlations were found between SoC-13 and several factors concerned with perceived wellbeing: perceived financial status, perceived social life, belief in the future, quality of life, perceived effect on mental health by the pandemic, perceived stress, anxiety, and depression. Noted are also significant inverse correlations to civil status and insomnia, but no correlations were found with sex, HCC, and education.

**Table 3:** Correlations between relevant variables and sense of coherence 13

**Variables**	***r*-value**	***p*-value**
Sex	-0.065	0.30
Civil status	-0.136	0.045*
Education	0.105	0.10
Perceived financial status	-0.164	0.009*
Perceived health	-0.235	< 0.001*
Perceives social life	-0.282	< 0.001*
Perceived insomnia	-0.135	0.03*
Belief in the future	-0.288	< 0.001*
Cortisol concentrations	0.020	0.75
EQ-5D	0.355	< 0.001*
Perceived negative effect on mental health due to the COVID -19 pandemic	-0.313	< 0.001*
PSS-10	-0.242	< 0.001*
HADS-A	-0.554	< 0.001*
HADS-D	-0.297	< 0.001*
GDS-20	-0.410	< 0.001*

*PSS-10* Perceived Stress Scale 10-item, *HADS-A* Hospital Anxiety and Depression Scale—Anxiety, *HADS-D* Hospital Anxiety and Depression Scale—Depression, *GDS-20* Geriatric Depression Scale 20-item

^*^significant difference

*r*-value measured by Spearman’s rho

As displayed in Table 4, linear regressions with SoC as the dependent variable were performed in a three-step approach: model 1 focusing on sociodemographic factors, model 2 adding factors relevant to general wellbeing, and model 3 completing it with factors concerning mental health. The third model explains up to 45% of the levels of SoC in the studied population (adjusted R square 0,449) and shows a significant inverse linear association with perceived financial status, belief in the future, perceived negative effect on mental health due to the COVID -19 pandemic, and anxiety, in such a way that low SoC correlates to poor wellbeing in each of the factors, and vice versa. These four variables were then analyzed in a final linear regression model, see Table S2, showing strengthening of the significances, and approximately the same level of explanation (adjusted R square 0.439) with anxiety as the strongest associated factor with SoC. The priori path model showed that the association with SoC for *mentally affected* mostly operated indirectly via anxiety (96%, *p* < 0.001), but mostly directly for *socially affected* (82%, *p* = 0.17), see Fig. 2.

**Table 4:** Three regression models containing all relevant variables with sense of coherence 13 as the dependent variable

**Variables**	**Model 1**			**Model 2**			**Model 3**		
	**Standardized coefficients beta**	**t**	***p*-value**	**Standardized coefficients beta**	**t**	***p*-value**	**Standardized coefficients beta**	**t**	***p*-value**
**Constant**	-	43.37	< 0.001	-	14.24	< 0.001	-	12.47	< 0.001
**Sex**	-0.054	-0.90	0.37	0.029	0.48	0.64	0.057	0.99	0.32
**Civil status**	-0.043	-0.70	0.49	-0.10	-1.65	0.10	-0.084	-1.52	0.13
**Education**	0.064	1.04	0.30	0.056	0.93	0.35	0.027	0.50	0.62
**Perceived financial status**	-0.14	-2.20	0.029*	-0.096	-1.55	0.12	-0.15	-2.68	0.008*
**Perceived social life**	-0.29	-4.64	< 0.001*	-0.13	-1.95	0.052	-0.12	-1.82	0.071
**EQ-5D-3L**	-	-	-	0.24	3.32	0.001*	-0.002	-0.03	0.98
**Perceived health**	-	-	-	-0.015	-0.22	0.83	0.051	0.78	0.43
**Perceived insomnia**	-	-	-	-0.061	-1.01	0.31	-0.01	-0.18	0.86
**Belief in the future**	-	-	-	-0.19	-2.90	0.004*	-0.15	-2.48	0.014*
**Cortisol logarithmic values**	-	-	-	-	-	-	0.009	0.17	0.87
**Perceived negative effect on mental health due to the COVID-19 pandemic**	-	-	-	-	-	-	-0.15	-2.60	0.010*
**PSS-10**	-	-	-	-	-	-	-0.045	-0.79	0.43
**HADS-A**	-	-	-	-	-	-	-0.47	-6.20	< 0.001*
**HADS-D**	-	-	-	-	-	-	-0.074	-1.26	0.21
**GDS-20**	-	-	-	-	-	-	0.002	0.025	0.98

*PSS-10* Perceived Stress Scale 10-item, *HADS-A* Hospital Anxiety and Depression Scale—Anxiety, *HADS-D* Hospital Anxiety and Depression Scale -Depression, *GDS-20* Geriatric Depression Scale 20-item

^*^significant difference

Regression Model 1: *p* < 0.001, df = 5, *F* = 6.975 (*R*^*2*^ 0.06)

Regression Model 2: *p* < 0.001, df = 9, *F* = 8.090 (*R*^*2*^ 0.238)

Regression Model 3: *p* < 0.001, df = 15, *F* = 12.015 (*R*^*2*^ 0.449)

## Discussion

In this older Swedish COVID -19 cohort, we observed a significant association between SoC and anxiety where low SoC related to more anxiety. This is coherent with previous findings of an inverted association between SoC and mental health [3, 32], and underlines the importance of society working to improve supportive measures. It is well known that anxiety is common in older adulthood, probably even underestimated since little is known about assessment of anxiety later in life [49], and adding to this underlying condition could be the context of living in a pandemic with fear of the virus, and social isolation [21, 50, 51]. During such conditions belief and hope for the future might be more difficult to sustain but hope has been described as a resilience factor when coping with stress, promoting wellbeing [52]. This present study revealed some increase towards a pessimistic belief in the future during the pandemic compared to pre-pandemic, but not significantly (data not shown). SoC has been associated with hope in older adults [53], and high SoC within families has been related to high hope and lower anxiety and depression [54]. This is consistent with the significant associations between SoC and belief in the future, respectively, anxiety in this study. Interestingly, depression does not show a likewise strong association with SoC as anxiety here, but a plausible explanation for this absence of association could be that the participants in this study were older people and for this group other factors could be more relevant. Anxiety and dejection are more predominant for this age-group rather than depression. However**,** there is an inverted relationship for both GDS 20 and HADS-D, which is in line with earlier findings [2, 55, 56]. Perceived negative effect on mental health due to the COVID -19 pandemic was, like anxiety, significantly associated with SoC. This variable was more deeply investigated in our previous study [57], showing that risk factors for being affected by the pandemic were, among other, anxiety, and experiencing a bad family situation and social life. It is therefore not surprising for this group to score low SoC, since healthy relationships and a good mental state are known to be of importance for developing and maintaining high SoC [2, 32]. A priori path model pointed towards the possibility that anxiety is important in explaining the association between those who perceived to be mentally affected by the pandemic and SoC. However, since the model is being estimated from a cross-sectional design it is important to note that causal interpretations cannot be made from it, but the findings are an important first step that should be followed up using a longitudinal design.

This study also found a significant association between SoC and perceived financial status. Antonovsky described that SoC can be molded by social structures [2]. Socioeconomic status is one such structure, and in this study, focus is on the perception of economic status rather than actual economic status. These two do not necessarily reflect one another, and the perceived aspect is rarely investigated even though one can presume that it might influence both mental health, and level of stress. There is also little knowledge about this perspective and SoC, even less so in the older population. There are two previous studies from Sweden and Canada on adults in working ages, and in both, income level was found to be related to SoC [55, 58], but none of them studied the perceived aspect of financial status. Thus, finding such an association between the perception of economic status and SoC in this study indicate the need for further research to better understand this relationship, as well as investigating and developing suitable social support systems, which was beyond the scope of this study.

No association was found between SoC and HCC in this study, and contradictive to previous research there were no significant differences in HCC between sexes. Actually, high cortisol levels were equally distributed between women and men. Women in this study suffered from more symptoms of anxiety and depression than men, which could indicate a more active HPA-axis, however, studies show that even though depression is associated with higher HCC, anxiety disorders appear to lower the cortisol levels [25].

Neither sex nor insomnia were associated with SoC, however, there were more women with lower quality of life, and higher scores for anxiety, depression, and stress in this study, which is in line with previous research [17, 18, 59, 60]. Research from the pandemic also suggests that women responded to the COVID -19 pandemic with more depressive symptoms than men [61, 62]. An explanation for this could be that women have been shown to be more emotionally affected by life stressors than men, which might also explain the slightly higher levels of HCC in women in this study. It has also been suggested that men and women use their coping resources differently [55, 63]. More knowledge is needed about these mechanisms to better understand how to support and improve coping skills in older men and women, both in general and during life changing events like the pandemic.

Noticeably, previous results from this study population showed that the majority of them lived in a relationship and displayed wellbeing [57]. Viewed from a salutogenic perspective, high SoC is here correlated to perceived good: mental health, general health, financial status, social life, and sleep. This could account for good coping, and, thus, lower risk for developing mental health issues when faced with a difficult life situation such as the COVID -19 pandemic [2, 3]. Important to note is also that older adults tend to have higher SoC than younger adults, i.e. better coping skills developed from life experiences [64]. This might have had a protective function during the pandemic against declined mental health and stress. However, the older adults were especially targeted by the COVID -19 pandemic, and therefore it is of importance to further understand what factors and social support (governmental, health care, communal etc.) that are important for sustaining wellbeing, and improve coping in the older population during such adverse exposure [65].

A major strength in this study is the focus on SoC and its association with mental health and HCC, a biological marker for HPA-axis activity. To our knowledge, no research has been published studying this in older adults, and only one study has been found investigating this in younger ages, finding evidence for associations between exposure to trauma, HPA-axis activity, and SoC [29]. In our present study no such association was found, perhaps due to a generally wellbeing population, but it still contributes to building knowledge, and, of course, more research of this kind is needed. Another strength is using a variety of reliable and well-validated screening scales measuring different aspects of mental health. Further strengthening the results are good agreement between them. The use of only one screening scale for anxiety, in comparison to two for depression, could be considered a limitation. However, HADS is considered a reliable tool to screen for anxiety, also in older adults, and as our results are rigid showing consistent statistical significance, we find them reliable and unlikely to change direction if another screening scale was used. A limitation is that we only have scores regarding stress, anxiety, and depression from the ongoing pandemic, and, thus, nothing to compare them to, so, at present, we do not know if the pandemic causes those scale values or not, but a follow-up study is planned. A well-known phenomenon to take into consideration when using self-reported data is the possible impact of recall bias, which could be a limitation. However, self-reports are in general considered well-established and reliable [66]. A limitation in all studies that use HCC is the difficulty of comparing these data to that from other research, since there are multiple methods of analyses that can give slightly varying results. Other limitations could be a gender bias, considering that, in general, women have more hair than men, and that some men are bold. In this study RIA was used for analysing HCC. This method can detect very small concentrations of cortisol and is little influenced by confounders like e.g. hair products, which can be consider a strength. For a broader discussion on the strengths and limitations of the HOLD-population we refer to our previous study [57]. Briefly, however, it is important to note a couple of limitations considering the recruitment process and study population. According to exclusion criteria, individuals living in nursing homes, not able to visit their PHCC, with cognitive dysfunction, and those with inadequate language skill were excluded. This, in combination with the well-known phenomenon that healthy people are more likely to participate in research than those in poor health means that frail elderly and immigrants were underrepresented in this study.

## Conclusion

Older adults from a primary care setting showed a significant association between SoC and anxiety, perceived economic status, belief in the future, and perceived negative mental effect from to the COVID -19 pandemic. The association with anxiety was the strongest, and anxiety was also suggested to be important in explaining the association between perceived mental effect due to the pandemic and SoC. Even though there was no association between SoC and sex, it is notable that women scored higher than men for symptoms of anxiety and depression, as well as for lower quality of life. HCC showed no significant association with SoC or sex, but in contrast to pre-pandemic studies high levels of HCC were evenly distributed between women and men. In clinical work, as well as in other social functions, the results indicate a need to pay special attention to the mental health and life quality of older women exposed to life changing events.

### Supplementary Information

Supplementary Material 1. 

Supplementary Material 2.

## Data Availability

No datasets were generated or analysed during the current study.

## Abbreviations

GDS 20: Geriatric Depression Scale 20 items
HADS: Hospital Anxiety and Depression Scale
HADS-A: Hospital Anxiety and Depression Scale – Anxiety
HADS-D: Hospital Anxiety and Depression Scale – Depression
HCC: Hair Cortisol Concentration
HOLD: Healthy OLD people
HPA axis: Hypothalamic-Pituitary-Adrenal axis
PHCC: Primary Health Care Center
PSS 10: Perceived Stress Scale 10 items
PTSD: Post Traumatic Stress Disorder
RIA: RadioImmunoAssay
SoC: Sense of Coherence
SoC-13: Sense of Coherence 13 items
