# Supplementary material for: Sense of coherence, mental health, and hair cortisol concentrations among older people during the COVID -19 pandemic: a cross-sectional study
Source: BMC Public Health. 2024 Jun 5;24:1502. doi: 10.1186/s12889-024-19034-3 (PMC11151583; doi:10.1186/s12889-024-19034-3)
Supplement: Supplementary file 1 — Supplementary Material 1. [file 12889_2024_19034_MOESM1_ESM.docx]

***Table S1.*** *Intercorrelations of HADS-D, HADS-A, GDS, EQ-5D, PSS-10 and SoC scales.*

| Scales | HADS-D | HADS-A | GDS-20 | EQ-5D | PSS-10 | SoC |
| --- | --- | --- | --- | --- | --- | --- |
|  | r p | r p | r p | r p | r p | r p |
| HADS-D | - | ,335 <,001 | ,271 <,001 | -,248 <,001 | ,073 ,24 | -,279 <,001 |
| HADS-A | ,335 <001 | - | ,610 <,001 | -,450 <,001 | ,281 <,001 | -,573 <,001 |
| GDS-20 | ,271 <,001 | ,610 <,001 | - | -,438 <,001 | ,181 ,004 | -,398 <,001 |
| EQ-5D | -,248 <,001 | -,450 <,001 | -,438 <,001 | - | ,055 ,25 | ,360 <,001 |
| PSS-10 | ,073 ,24 | ,281 <,001 | ,181 ,004 | ,055 ,25 | - | -,233 <,001 |
| SoC | -,279 <,001 | -,573 <,001 | -,398 <,001 | ,360 <, 001 | -,233 <,001 | - |
