# Supplementary material for: Sense of coherence, mental health, and hair cortisol concentrations among older people during the COVID -19 pandemic: a cross-sectional study
Source: BMC Public Health. 2024 Jun 5;24:1502. doi: 10.1186/s12889-024-19034-3 (PMC11151583; doi:10.1186/s12889-024-19034-3)
Supplement: Supplementary file 2 — Supplementary Material 2. [file 12889_2024_19034_MOESM2_ESM.docx]

***Table S2.*** *Regression model with variables falling out as statistically significant in the three-model regression.*

| **Variables** | **Standardized Coefficients Beta** | **t** | **p-value** |
| --- | --- | --- | --- |
| **Constant** | n/a | 106.11 | <0.001* |
| **Perceived financial status** | -0.15 | -3.01 | 0.003* |
| **Belief in the future** | -0.18 | -3.24 | 0.001* |
| **Perceived negative affect on mental health due to the COVID -19 pandemic** | -0.17 | -3.17 | 0.002* |
| **HADS-A** | -0.49 | -8.79 | <0.001* |

*Sense of Coherence as dependent variable.*

*HADS-A (Hospital Anxiety and Depression Scale - Anxiety)*

**significant difference*

*p<0.001, df=4, F=43.915*
